# Supplementary material for: Unravelling raked linear dunes to explain the coexistence of bedforms in complex dunefields
Source: Nat Commun. 2017 Jan 27;8:14239. doi: 10.1038/ncomms14239 (PMC5290144; doi:10.1038/ncomms14239)
Supplement: Supplementary Information — Supplementary Figures. [file ncomms14239-s1.pdf]

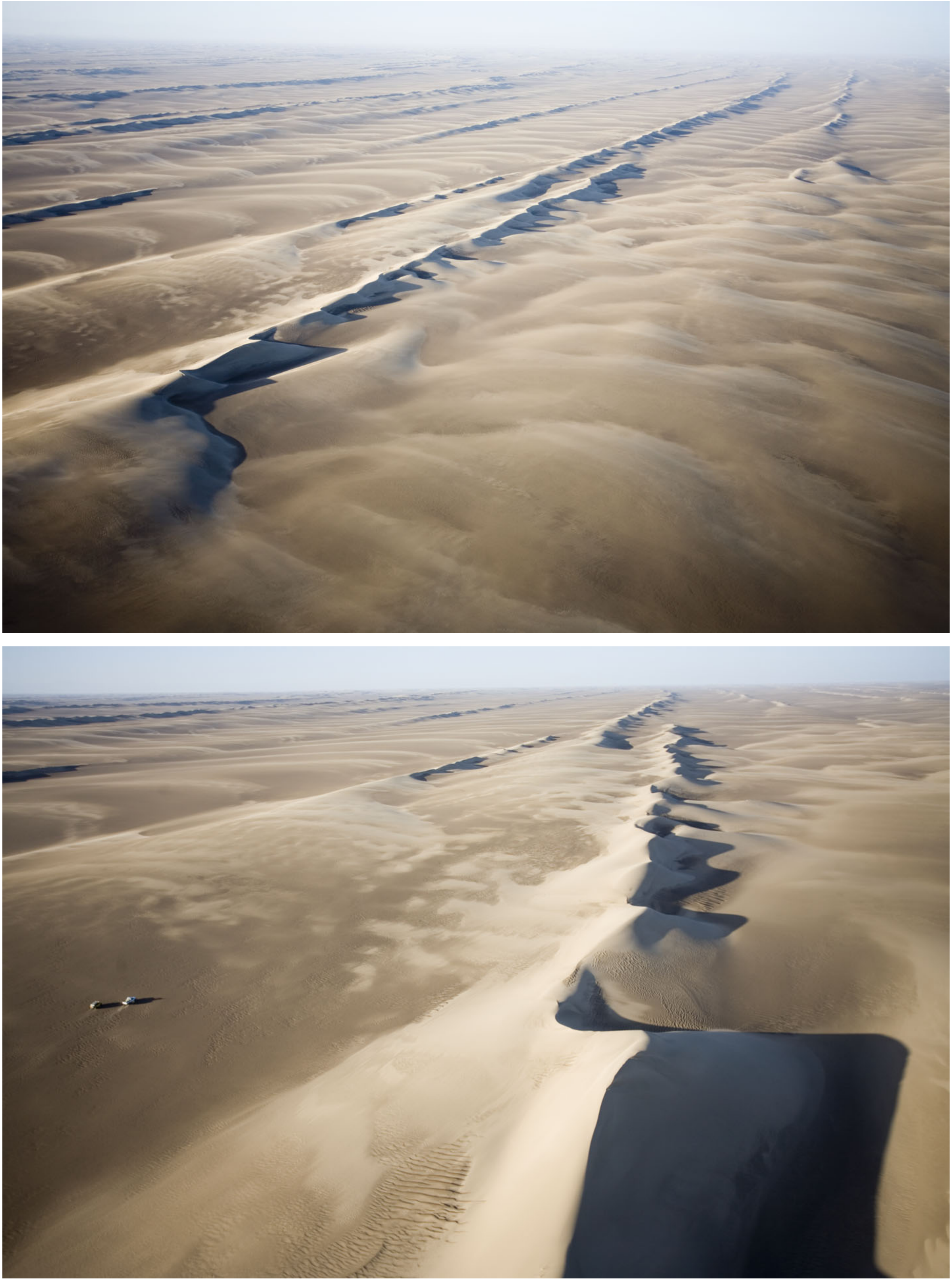

Figure Supplementary 1: Aerial views of the raked linear dunes in the Kumtagh desert (courtesy of George Steinmetz). Cars in the bottom picture are for scale. Note the marked asymmetry between a smooth undulating slope on the left side of the linear dunes and a periodic pattern of secondary ridges on the right side.

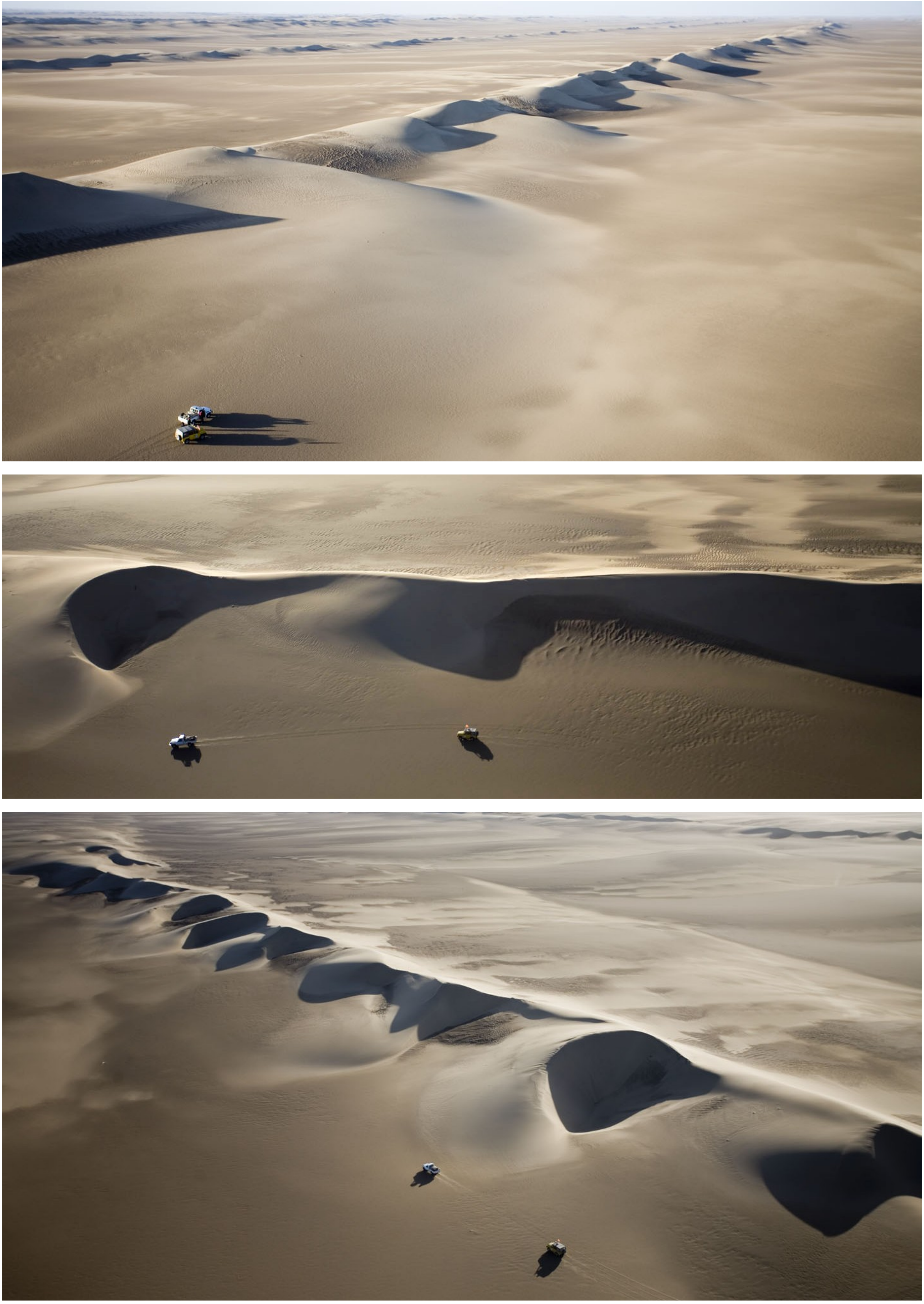

Figure Supplementary 2: Aerial views of the raked linear dunes in the Kumtagh desert (courtesy of George Steinmetz). Cars are for scale. The top picture is taken looking in the direction of elongation. The bottom picture is taken in the opposite direction. The middle picture is taken perpendicularly to the primary ridge.

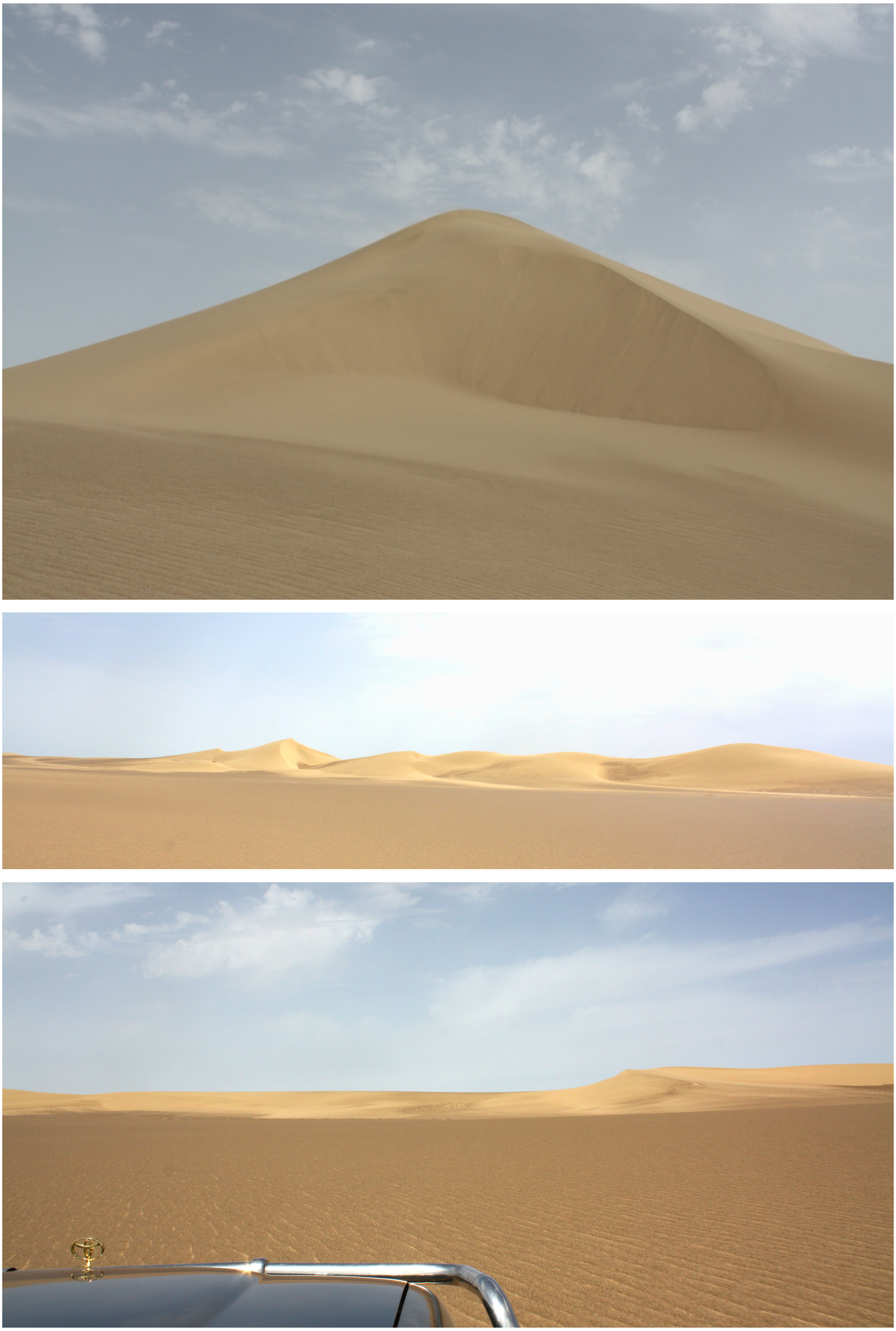

Figure Supplementary 3: Ground views of raked linear dunes. (top) Slip faces with different orientations at the crest of a superimposed bedform. (middle and bottom) Side views of a raked linear dune.

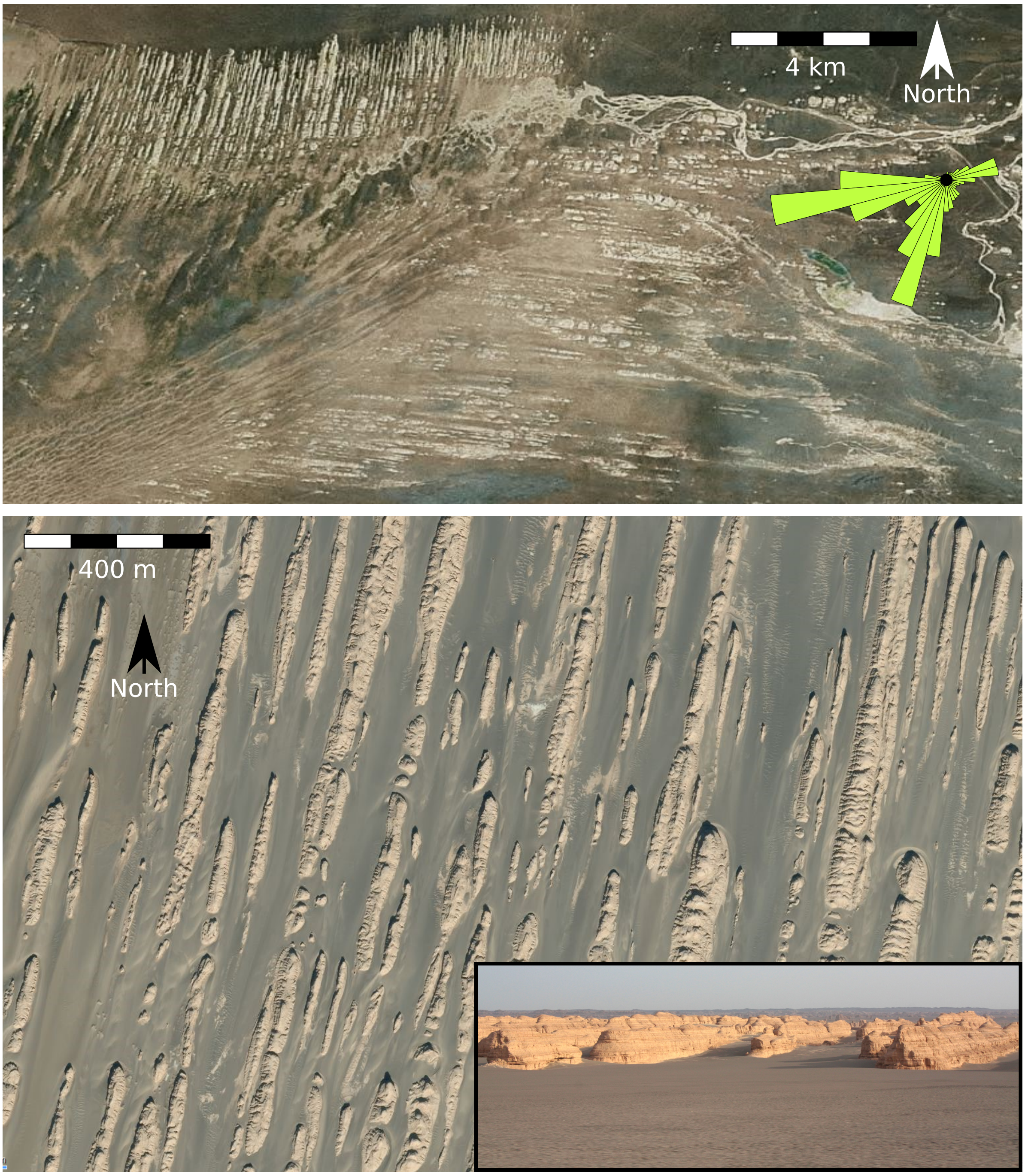

Figure Supplementary 4: Yardangs at the northeast end of the Kumtagh desert: (top) satellite view of the Yardang National Geological Park located where the raked linear dunes start to elongate; (Bottom) aerial and side views of the yardangs with a north-south alignment. The inset shows the sand flux rose in the raked linear dunefield, 30 km east from the yardangs. The general agreement between the principal sand flux orientations and the yardang alignments suggests strong similarities between the modern winds and the wind regime that gave rise to the yardangs in the past.

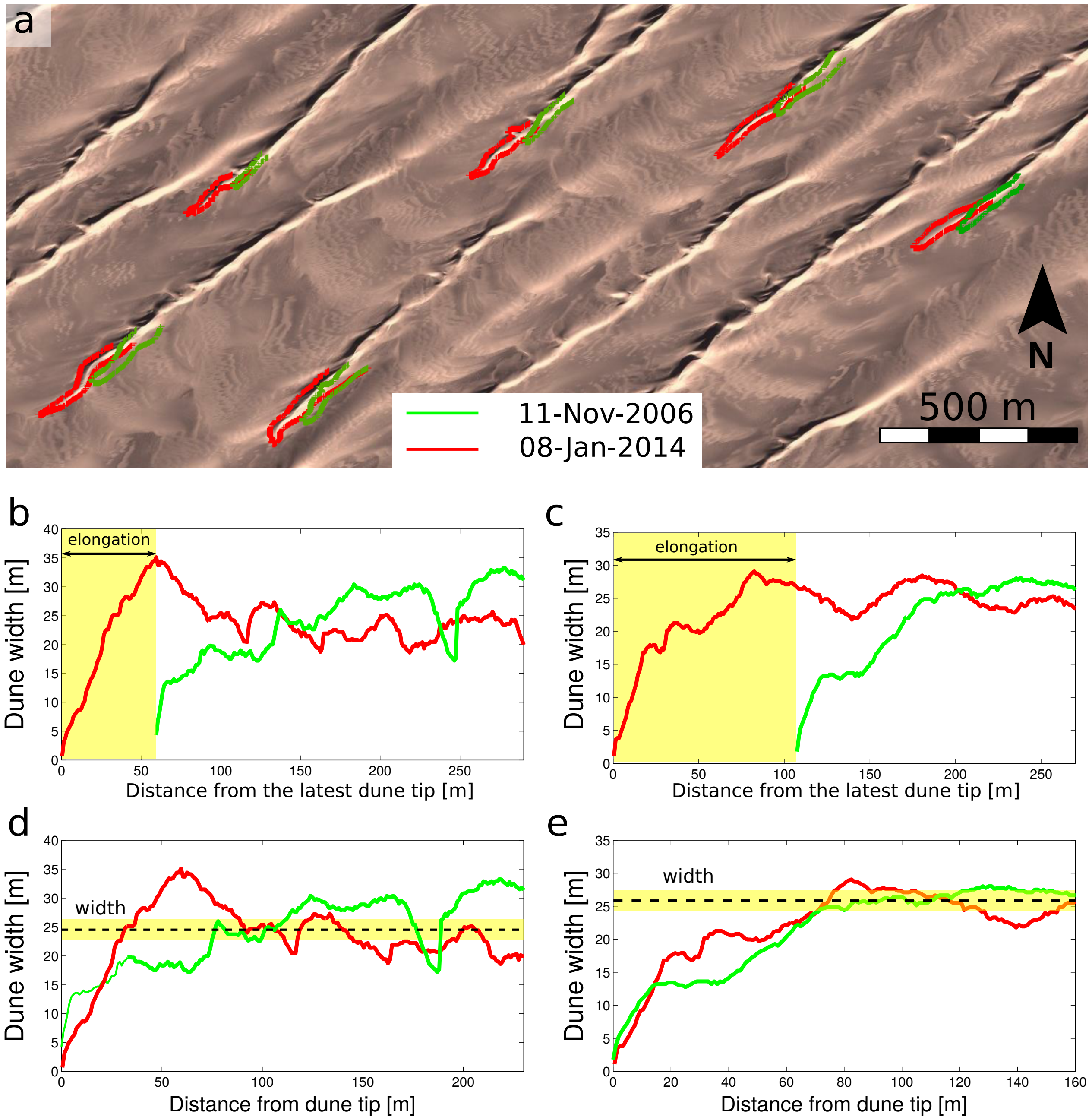

Figure Supplementary 5: Elongation and mean width of linear dunes in the Kumtagh desert (92°13′ East, 40°14′ North). (a) Individual contours of the growing tips of linear dunes at two different times: 11th of October, 2006 (green); 8th of January, 2014 (red). (b-c) Dune width with respect to the distance from the latest dune tip for two linear dunes. These widths are measured perpendicularly to the linear dune orientations, which are computed from the surface area of these dunes. Elongation estimates are the distance between the dune tips at two different times (double arrows). (d-e) Dune width with respect to the distance from dune tip. The mean dune width of the growing dune is the steady-state width reached along the dune body away from the growing tip (dashed lines). Note the change in dune shape during dune growth.

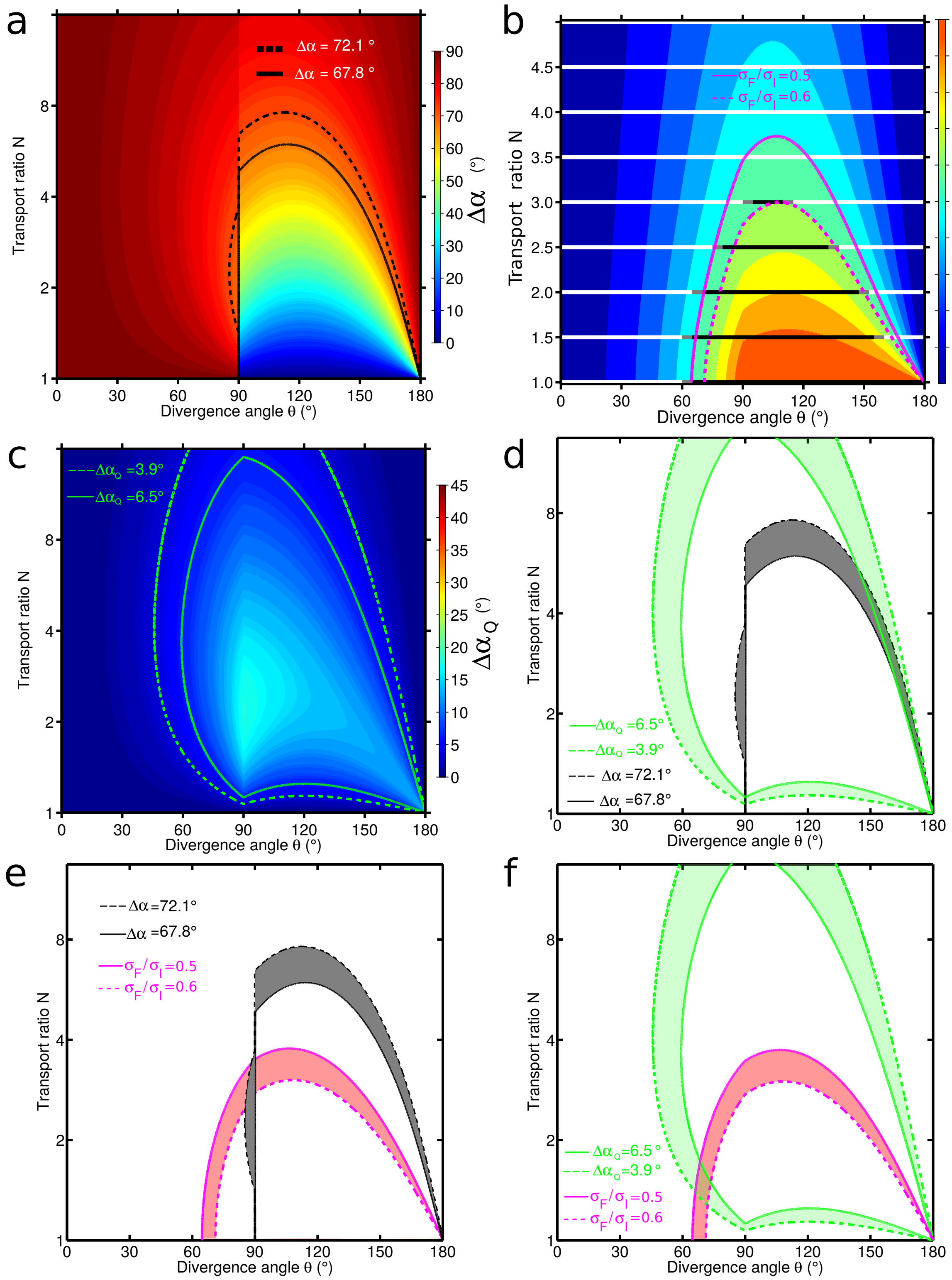

Figure Supplementary 6: Dune properties in the parameter space $\{\theta, N\}$ of bidirectional wind regimes using a flux-up ratio $\gamma = 1.6$. θ and N are the angle and the transport ratio between the two winds, respectively. **(a)** Angle $\Delta\alpha$ between dune crest orientations in the bed instability and fingering modes. **(b)** Growth rate ratio $\sigma_{\mathrm{F}}/\sigma_{\mathrm{I}}$ between the two dune growth mechanisms. Black and white lines show the zones of the parameter space in which linear dunes and train of barchan dunes are observed, respectively. Gray lines indicate transition zones where both dune patterns coexist. No raked-linear dunes are observed. **(c)** Angle $\Delta\alpha_{\mathrm{Q}}$ between the sand flux directions at the crest of dunes in the bed instability and fingering modes. In (a), (b) and (c) contour lines show the values predicted from the winds recorded by the wind tower W_1 (solid) and W_2 (dashed). **(d-f)** Comparisons between the values predicted from the wind data in the parameter space $\{\theta, N\}$ of bidirectional wind regime: **(d)** comparisons between the $\Delta\alpha$ and $\Delta\alpha_{\mathrm{Q}}$-values. **(e)** comparisons between the $\Delta\alpha$ and $\sigma_{\mathrm{F}}/\sigma_{\mathrm{I}}$-values. **(f)** comparisons between the $\Delta\alpha_{\mathrm{Q}}$ and $\sigma_{\mathrm{F}}/\sigma_{\mathrm{I}}$-values.
